# Pregnancy intention data completeness, quality and utility in population-based surveys: EN-INDEPTH study

**DOI:** 10.1186/s12963-020-00227-y

**Published:** 2021-02-08

**Authors:** Judith Yargawa, Kazuyo Machiyama, Victoria Ponce Hardy, Yeetey Enuameh, Edward Galiwango, Kassahun Gelaye, Kaiser Mahmud, Sanne M. Thysen, Damazo T. Kadengye, Vladimir Sergeevich Gordeev, Hannah Blencowe, Joy E. Lawn, Angela Baschieri, John Cleland, the Every Newborn-INDEPTH Study Collaborative Group, Peter Byass, Joy E. Lawn, Peter Waiswa, Hannah Blencowe, Judith Yargawa, Joseph Akuze, Ane B. Fisker, Justiniano S. D. Martins, Amabelia Rodrigues, Sanne M. Thysen, Gashaw Andargie Biks, Solomon Mokonnen Abebe, Tadesse Awoke Ayele, Telake Azale Bisetegn, Tadess Guadu Delele, Kassahun Alemu Gelaye, Bisrat Misganaw Geremew, Lemma Derseh Gezie, Tesfahun Melese, Mezgebu Yitayal Mengistu, Adane Kebede Tesega, Temesgen Azmeraw Yitayew, Simon Kasasa, Edward Galigawango, Collins Gyezaho, Judith Kaija, Dan Kajungu, Tryphena Nareeba, Davis Natukwatsa, Valerie Tusubira, Yeetey A. K. Enuameh, Kwaku P. Asante, Francis Dzabeng, Seeba Amenga Etego, Alexander A. Manu, Grace Manu, Obed Ernest Nettey, Sam K. Newton, Seth Owusu-Agyei, Charlotte Tawiah, Charles Zandoh, Nurul Alam, Nafisa Delwar, M. Moinuddin Haider, Md. Ali Imam, Kaiser Mahmud, Angela Baschieri, Simon Cousens, Vladimir S. Gordeev, Victoria Ponce Hardy, Doris Kwesiga, Kazuyo Machiyama

**Affiliations:** 1 Maternal, Adolescent, Reproductive & Child Health (MARCH) Centre, London School of Hygiene & Tropical Medicine, London, UK; 2 Kintampo Health Research Centre, Kintampo, Ghana; 3 Department of Epidemiology and Biostatistics, Kwame Nkrumah University of Science and Technology, Kumasi, Ghana; 4 IgangaMayuge Health and Demographic Surveillance System, Makerere University Centre for Health and Population Research, Makerere, Uganda; 5 Dabat Research Centre Health and Demographic Surveillance System, Dabat, Ethiopia; 6 Department of Epidemiology and Biostatistics, Institute of Public Health, University of Gondar, Gondar, Ethiopia; 7 Health Systems and Population Studies Division, icddr,b, Dhaka, Bangladesh; 8 Bandim Health Project, Bissau, Guinea-Bissau; 9 Research Centre for Vitamins and Vaccines, Statens Serum Institut, Copenhagen, Denmark; 10 Department of Clinical Research Open Patient data Explorative Network (OPEN), University of Southern Denmark, Odense, Denmark; 11 Data, Measurement and Evaluation Unit, African Population and Health Research Centre, Nairobi, Kenya; 12 Institute of Population Health Sciences, Queen Mary University of London, London, UK

**Keywords:** Pregnancy intention, Fertility, Measurement, Assessment, Survey, Retrospective reporting, Desired family size, Stillbirth, Neonatal mortality, Low birthweight

## Abstract

**Background:**

An estimated 40% of pregnancies globally are unintended. Measurement of pregnancy intention in low- and middle-income countries relies heavily on surveys, notably Demographic and Health Surveys (DHS), yet few studies have evaluated survey questions. We examined questions for measuring pregnancy intention, which are already in the DHS, and additional questions and investigated associations with maternity care utilisation and adverse pregnancy outcomes.

**Methods:**

The EN-INDEPTH study surveyed 69,176 women of reproductive age in five Health and Demographic Surveillance System sites in Ghana, Guinea-Bissau, Ethiopia, Uganda and Bangladesh (2017–2018). We investigated responses to survey questions regarding pregnancy intention in two ways: (i) pregnancy-specific intention and (ii) desired-versus-actual family size. We assessed data completeness for each and level of agreement between the two questions, and with future fertility desire. We analysed associations between pregnancy intention and number and timing of antenatal care visits, place of delivery, and stillbirth, neonatal death and low birthweight.

**Results:**

Missing data were <2% in all questions. Responses to pregnancy-specific questions were more consistent with future fertility desire than desired-versus-actual family size responses. Using the pregnancy-specific questions, 7.4% of women who reported their last pregnancy as unwanted reported wanting more children in the future, compared with 45.1% of women in the corresponding desired family size category. Women reporting unintended pregnancies were less likely to attend 4+ antenatal care visits (aOR 0.73, 95% CI 0.64–0.83), have their first visit during the first trimester (aOR 0.71, 95% CI 0.63–0.79), and report stillbirths (aOR 0.57, 95% CI 0.44–0.73) or neonatal deaths (aOR 0.79, 95% CI 0.64–0.96), compared with women reporting intended pregnancies. We found no associations for desired-versus-actual family size intention.

**Conclusions:**

We found the pregnancy-specific intention questions to be a much more reliable assessment of pregnancy intention than the desired-versus-actual family size questions, despite a reluctance to report pregnancies as unwanted rather than mistimed. The additional questions were useful and may complement current DHS questions, although these are not the only possibilities. As women with unintended pregnancies were more likely to miss timely and frequent antenatal care, implementation research is required to improve coverage and quality of care for those women.

## Key findings

**Table:** 

**What is new?**	
• **What was known already**: Surveys, notably Demographic Health Surveys (DHS) are the major source of data on pregnancy intention in low- and middle-income countries; however, few studies have evaluated the actual questions used. • **What was done:** The EN-INDEPTH study in five Health and Demographic Surveillance System sites in Ghana, Guinea-Bissau, Ethiopia, Uganda and Bangladesh surveyed 69,176 women of reproductive age, providing an opportunity to examine pregnancy intention questions for data quality and utility. Two sets of questions used in DHS regarding pregnancy intention (pregnancy-specific intention and desired-versus-actual family size) were evaluated for completeness and data quality. Associations between pregnancy intention and maternal health care utilisation, and adverse pregnancy outcomes were also assessed.	
**What was found?**	
• **Completeness of responses:** Missing data were <2% in all questions. For desired-versus-actual family size questions, there was a relatively high proportion of ‘don’t know’ responses (≥10%) notably for questions requiring a numerical response. Consequently, 12.1% of responses had to be excluded from the desired-versus-actual family size assessment. • **Data quality (level of agreement):** The desired-versus-actual family size assessment was inconsistent with future fertility desire. A total of 7.4% of women reported their most recent pregnancy as unwanted but reported wanting more children in the future using the pregnancy-specific questions, compared with 45.1% in the corresponding category of the desired-versus-actual family size assessment. • **Prevalence estimates:** Using the pregnancy-specific questions, prevalence of unwanted pregnancy was 4.1% and unintended pregnancy 19.5% (unwanted plus mistimed pregnancies). Prevalence of undesired pregnancy was 25.2% using the desired-versus-actual family size. • **Data utility:** Using the pregnancy-specific questions, women with unintended pregnancies were less likely to have four ANC visits, start ANC in the first trimester, and report stillbirths and neonatal deaths. We found no association with facility birth and low birthweight. We found no association between desired-versus-actual family size and any of these outcomes.	
**What next in measurement and research?**	
• **Measurement improvement now:** The pregnancy-specific intention questions, which had binary/categorical response options, had higher completeness and level of agreement with future fertility desire than the desired-versus-actual family size assessment. The additional questions might complement current survey questions, although these are not the only possible additions to the standard DHS approach. Research studies need to consider the challenges of asking women to provide numeric responses regarding desired family size. • **Research needed:** o Since surveys remain the main data source for measuring pregnancy intention in low- and middle- income countries, it is important to advance methods for prospectively establishing intention prior to pregnancy. o Reluctance of women with unintended pregnancies to report stillbirths and neonatal deaths in surveys requires further investigation. o Women with unintended pregnancies may already be vulnerable and we found that they are more likely to miss timely and frequent antenatal care, so implementation research is required as to how to improve coverage and quality of care for those women.	

## Background

Worldwide, around 40% of pregnancies are estimated to be unintended, with high levels in low- and middle-income countries (LMICs) and high-income countries (HICs) [1, 2]. Previous published reviews have found associations between unintended pregnancy and adverse outcomes for both mother and newborn, including stillbirth, neonatal mortality, low birthweight and preterm birth [3–6]. There is also some evidence to suggest that women experiencing an unintended pregnancy may alter their health-seeking behaviours by delaying or reducing antenatal and postnatal care-seeking and increasing delivery at home, elevating risk of adverse outcomes for both women and their babies [4, 7–9]. However, the evidence base is mixed, and very few studies have examined the association between pregnancy intention and stillbirth or neonatal mortality [7, 8, 10–17]. Additionally, most studies have focused on HICs.

**Fig. 1:**
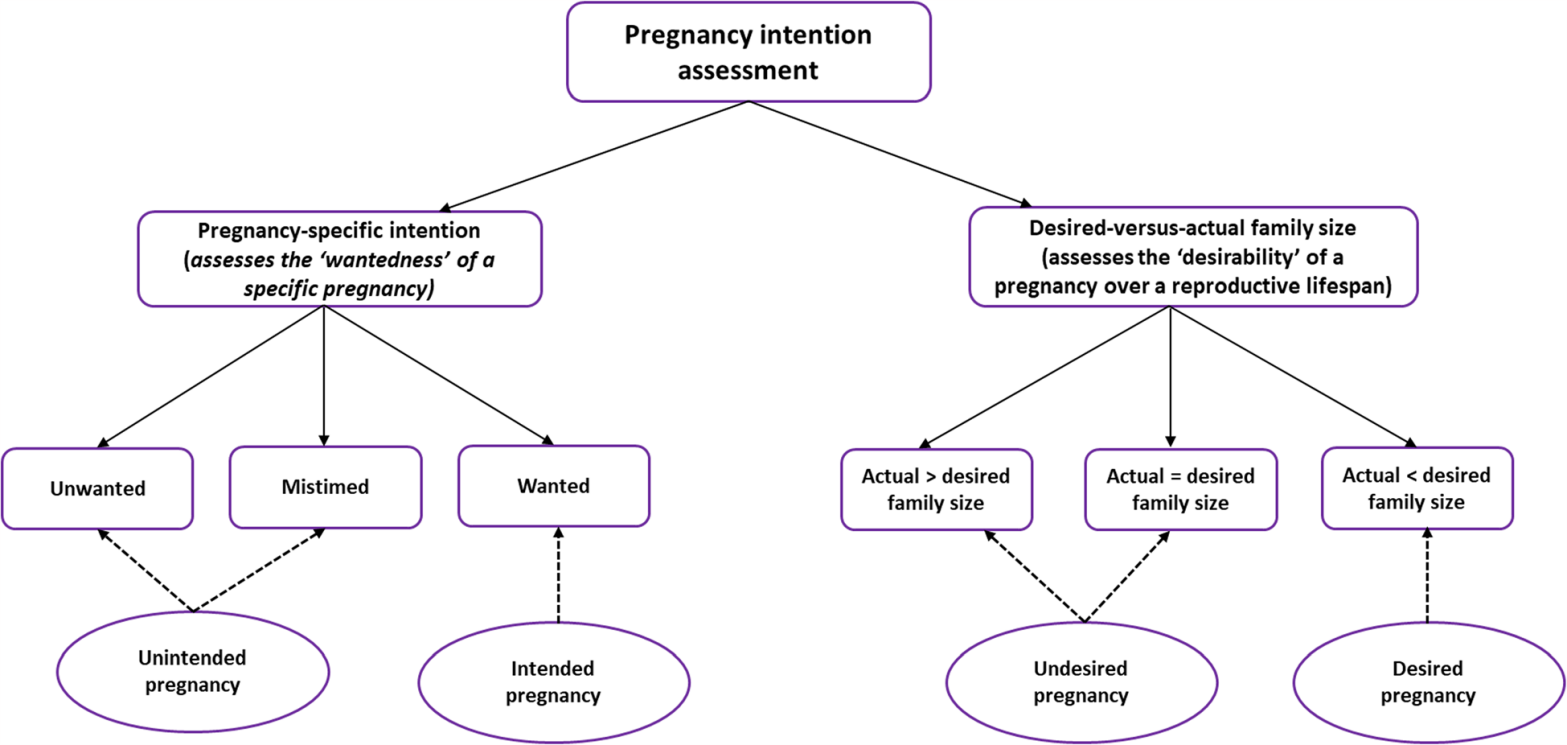
Overview of pregnancy intention assessment categories (Note: The desired-versus-actual family size assesses family size at the conception of the index pregnancy)

Most data worldwide on pregnancy intention are derived from cross-sectional household surveys. Questions in these surveys rely on a woman’s retrospective responses at interview, that is, after the pregnancy, regarding her intention—wanted, mistimed or unwanted—prior to conception (referred to here as *pregnancy-specific intention*). An alternative way of assessing pregnancy intention in surveys is to compare desired family size with actual number of surviving children; any pregnancy that occurs after the desired size has been reached is defined as undesired (referred to here as *desired-versus-actual family size*) (Fig. 1)*.* Both methods are widely used notably in the Demographic and Health Surveys (DHS) which have been undertaken in more than 90 countries [14, 18]. However, very few studies have comprehensively assessed these methods, including the level of agreement between them. In their study of pregnancy intention, Yeatman and Sennott assessed prevalence of unwanted and unintended pregnancies among young women in Malawi using seven measurement approaches, including both methods mentioned here; however, the level of agreement between these two approaches was not directly assessed [18].

This paper is part of a series of papers from the Every Newborn- International Network for the Demographic Evaluation of Populations and their Health (EN-INDEPTH) study in five Health and Demographic Surveillance System (HDSS) sites in sub-Saharan Africa and Asia. In this study, we compare *pregnancy-specific intention* and *desired-versus-actual family size* methods of assessing pregnancy intention to determine their level of agreement and utility for assessing pregnancy intention (Table 1A). We also investigate the level of agreement with, and utility of, additional questions on pregnancy intention (Table 1B), and associations between pregnancy intention, maternal health care utilisation, and adverse maternal and newborn outcomes.

**Table 1A:** EN-INDEPTH survey questions on pregnancy intention from DHS-7 and overview of responses

Domain	Question	Responses	Details	No. of potential respondents	No. of missing data *n* (%)	No. of don’t knows *n* (%)	No. of ‘other’ responses *n* (%)
Existing pregnancy-specific intention questions	1) ‘When you got pregnant with THIS BABY, did you want to get pregnant at that time?’	- Yes - No	Asked for last stillbirth, last neonatal death and last live birth survivor to D28 from the Full Birth History module only.	14,991	7 (0.05)	na	na
	2a) ‘Did you want to have a baby later on, or did you not want any children?’	- Later - No more/none	Asked to women who responded with ‘no’ in 1 and who had only ever given birth once.	705	10 (1.4)	na	na
	2b) ‘Did you want to have a baby later on, or did you not want any more children?’	- Later - No more/none	Asked to women who responded with ‘no’ in 1 and who had given birth more than once previously	2040	26 (1.3)	na	na
	3) How much longer did you want to wait?	- Months (if < 1 year) - Years (if > 12 months) - Don’t know	Asked to women who responded with ‘later’ in 2a and 2b. Not used in the calculation of pregnancy intention.	2173	37 (1.7)	331 (15.2)	na
Desired family size questions	4a) If you could go back to the time you did not have any children and could choose exactly the number of children to have in your whole life, how many would that be?	- None - Number (integer recorded) - Other (specified)	Asked to all women who had at least one child at the time of the interview. This section was excluded in Matlab.	11,493	59 (0.5)	1225 (10.7). The ‘other’ category included ‘don’t know’ responses	
	4b) If you could choose exactly the number of children to have in your whole life, how many would that be?	- None - Number (integer recorded) - Other (specified)	Asked to all women who had no children at the time of the interview. This section was excluded in Matlab.	109		13 (11.9). The ‘other’ category included ‘don’t know’ responses	

Numbers in table are unweighted

*na* not applicable

**Table 1B:** EN-INDEPTH survey questions on pregnancy intention from additional sources and overview of responses

Domain	Question	Responses	Details	No. of potential respondents	No. of missing data *n* (%)	No. of don’t knows *n* (%)
Additional questions on intention of the last pregnancy outcomes	1a) Right before you got pregnant with THIS BABY, how important was it to you to avoid/delay the pregnancy? Would you say very important, somewhat important or not at all important?	- Very important - Somewhat important - Not at all important	Asked to women who responded with ‘no’ in 1 in Table 1A	2745	7 (0.3)	na
	1b) Right before you got pregnant with THIS BABY, were you doing something to avoid or delay the pregnancy?	- Yes - No	Asked to women who responded with ‘no’ in 1 in Table 1B	2745	7 (0.3)	na
	1c) What were you doing?	Various contraception methods, with ‘other’ and ‘don’t know/ unsure’ options	All options that applied were ticked. Asked to women who responded with ‘yes’ in 1b above and ‘no’ in 1 in Table 1B	1083	0 (0.0)	2 (0.2)
	1d) When you found out you were pregnant with THIS BABY, did you consider or not consider terminating the pregnancy?	- Considered - Not considered	Asked to women who responded with ‘no’ in 1 in Table 1B	2745	7 (0.3)	na
Future fertility preference questions	2a) Now I have some questions about the future. Would you like to have (a/another) child, or would you prefer not to have any (more) children?	- Have (a/another) child - No more/none - Cannot get pregnant - Undecided/don’t know	Asked to women who were not pregnant at the time of the survey. This section was excluded in Matlab.	11,602	116 (1.0)	927 (8.0)
	2b) Now I have some questions about the future. After the child you are expecting now, would you like to have another child, or would you prefer not to have any more children?	- Have another child - No more - Undecided/don’t know	Asked to women who were pregnant at the time of the survey. This section was excluded in Matlab.			

Numbers in table are unweighted

*na* not applicable.

**Table 2:** Maternal characteristics of weighted pregnancy events from the five EN-INDEPTH HDSS sites (*n* = 14,984)

	Bandim *n* = 2708 *n* (%)	Dabat *n* = 2019 *n* (%)	IgangaMayuge *n* = 1975 *n* (%)	Kintampo *n* = 3625 *n* (%)	Matlab *n* = 4656 *n* (%)	Overall *n* = 14,984 *n* (%)
Maternal age at interview						
15–24	712 (26.3)	389 (19.3)	496 (25.1)	554 (15.3)	1478 (31.7)	3629 (24.2)
25–34	1349 (49.8)	975 (48.3)	985 (49.9)	1710 (47.2)	2545 (54.7)	7565 (50.5)
35+	647 (23.9)	655 (32.4)	494 (25.0)	1360 (37.5)	633 (13.6)	3789 (25.3)
Mean age	29.5	30.8	29.7	32.2	27.8	29.8
Marital status at interview^a^						
Currently in union	2108 (77.9)	1908 (94.5)	1797 (91.0)	3325 (91.7)	4656 (100)	13,794 (92.1)
Not in union	597 (22.1)	111 (5.5)	178 (9.0)	301 (8.3)	0 (0.0)	1187 (7.9)
Highest level of education						
No education	950 (35.1)	1313 (65.0)	170 (8.6)	1499 (41.3)	175 (3.8)	4108 (27.4)
Primary	746 (27.5)	434 (21.5)	1084 (54.9)	1800 (49.6)	822 (17.7)	4886 (32.6)
Secondary+	1012 (37.4)	272 (13.4)	720 (36.5)	327 (9.0)	3659 (78.6)	5990 (40.0)
Wealth quintile						
Poorest	690 (25.5)	263 (13.0)	272 (13.8)	716 (19.8)	887 (19.0)	2827 (18.9)
Poor	528 (19.5)	336 (16.7)	353 (17.9)	761 (21.0)	977 (21.0)	2956 (19.7)
Middle	469 (17.3)	447 (22.1)	365 (18.5)	704 (19.4)	924 (19.8)	2909 (19.4)
Rich	509 (18.8)	390 (19.3)	458 (23.2)	713 (19.7)	932 (20.0)	3003 (20.0)
Richest	512 (18.9)	582 (28.8)	528 (26.7)	731 (20.2)	936 (20.1)	3288 (21.9)
Number of living children at conception						
0	544 (20.1)	246 (12.2)	251 (12.7)	429 (11.8)	1308 (28.1)	2777 (18.5)
1–2	1277 (47.2)	690 (34.2)	660 (33.4)	1411 (38.9)	2971 (63.8)	7009 (46.8)
3–4	650 (24.0)	583 (28.9)	517 (26.2)	1138 (31.4)	351 (7.5)	3239 (21.6)
5+	237 (8.8)	500 (24.8)	548 (27.7)	647 (17.9)	26 (0.6)	1959 (13.1)
Mean no of living children	2.0	3.0	3.2	2.7	1.1	2.2
No of desired family size^a^						
0–2	436 (16.4)	618 (30.6)	144 (7.3)	483 (13.3)	na	1681 (16.4)
3–4	1155 (43.4)	477 (23.7)	570 (28.9)	1101 (30.4)	na	3303 (32.1)
5–6	393 (14.8)	420 (20.8)	850 (43.1)	1077 (29.7)	na	2740 (26.7)
7+	124 (4.7)	270 (13.4)	388 (19.7)	526 (14.5)	na	1309 (12.7)
Non-numeric	552 (20.7)	232 (11.5)	22 (1.1)	436 (12.0)	na	1241 (12.1)
Mean desired family size	3.8	3.6	5.4	4.6	na	4.4
Future fertility desire						
Want more	1864 (69.5)	1327 (65.7)	1354 (69.4)	2418 (67.7)	na	6963 (68.1)
No more	407 (15.2)	539 (26.7)	469 (24.0)	790 (22.1)	na	2205 (21.6)
Cannot get pregnant	2 (0.1)	5 (0.3)	43 (2.2)	26 (0.7)	na	77 (0.8)
Undecided/ don’t know	407 (15.2)	147 (7.3)	86 (4.4)	336 (9.4)	na	977 (9.6)
Median time since last delivery (months)						
	25	26	22	27	29	26
Number of ANC visits						
0	34 (1.4)	663 (32.9)	18 (0.9)	59 (2.1)	298 (6.4)	1072 (7.7)
1–3	424 (17.9)	716 (35.5)	642 (32.7)	519 (18.1)	1626 (35.0)	3927 (28.3)
4+	1910 (80.7)	636 (31.6)	1304 (66.4)	2287 (79.8)	2727 (58.6)	8865 (63.9)
Place of last delivery						
Home	797 (34.0)	1185 (59.7)	175 (9.4)	1158 (32.4)	1402 (39.6)	4718 (35.4)
Health facility	1549 (66.0)	801 (40.3)	1698 (90.6)	2416 (67.6)	2143 (60.4)	8606 (64.6)
Last pregnancy outcome						
Stillbirth	51 (1.9)	18 (0.9)	15 (0.7)	59 (1.6)	79 (1.7)	221 (1.5)
Neonatal death	99 (3.7)	52 (2.6)	56 (2.9)	81 (2.3)	101 (2.2)	390 (2.6)
Post-neonatal survivors	2559 (96.3)	1949 (97.4)	1904 (97.1)	3486 (97.7)	4476 (97.8)	14,373 (97.4)
Birthweight of last pregnancy outcome^b^						
< 2.5 Kg	203 (11.9)	21 (8.4)	156 (9.7)	105 (6.8)	626 (22.0)	1111 (13.9)
≥ 2.5 Kg	1501 (88.1)	233 (91.6)	1457 (90.3)	1448 (93.2)	2223 (78.0)	6862 (86.1)

Weighted numbers (‘*n*’) were rounded to the nearest whole numbers; hence, there may be slight variations in cumulative totals

*na* not applicable

^a^ Current marital status and also desired family size were not collected in Matlab

^b^ A total of 46.8% of the birthweight data were missing

This paper has two objectives:

1. ***Survey question performance:*** Evaluate the completeness of and level of agreement between (i) existing questions (Table 1A) and (ii) additional questions for assessing pregnancy intention (Table 1B)
2. ***Data utility:*** Assess the associations between unintended pregnancy and (i) maternal health care utilisation (number of antenatal care (ANC) visits, timing of first ANC visit and place of delivery) and (ii) adverse pregnancy outcomes (stillbirths, neonatal deaths and low birthweight), to inform programmatic action.

## Methods

### EN-INDEPTH study design and settings

The EN-INDEPTH study was a cross-sectional study of 69,176 women of reproductive age undertaken between August 2017 and August 2018 in five HDSS sites: Bandim in Guinea-Bissau, Dabat in Ethiopia, IgangaMayuge in Uganda, Matlab in Bangladesh and Kintampo in Ghana (Additional file 1 provides background details of these sites). Overall, the study aimed to inform improvements in the measurement of pregnancy outcomes in population-based household surveys, and its primary objective was to compare two methods of retrospective recording of pregnancy outcomes: a full birth history with additional questions on pregnancy losses (FBH+) and a full pregnancy history (FPH). The study also investigated the performance of existing and/or modified survey questions regarding other pregnancy-related outcomes, and conducted qualitative research exploring barriers and enablers to reporting these outcomes (Additional file 2). Further details have been published in the protocol and main study paper [19, 20]. The survey questions were administered face-to-face, and data were collected on tablets using the Survey Solutions system [21]. Interviewers were recruited locally, and hence knew the local language and culture. Data from all five sites were anonymised by local HDSS scientists upon completion of the data collection, encrypted and then shared. Data analyses and management were conducted using Stata version 15.1. Results are reported in accordance with STROBE Statement checklists for cross-sectional studies [22] (Additional file 3).

### EN-INDEPTH survey questions on pregnancy intention

**Fig. 2:**
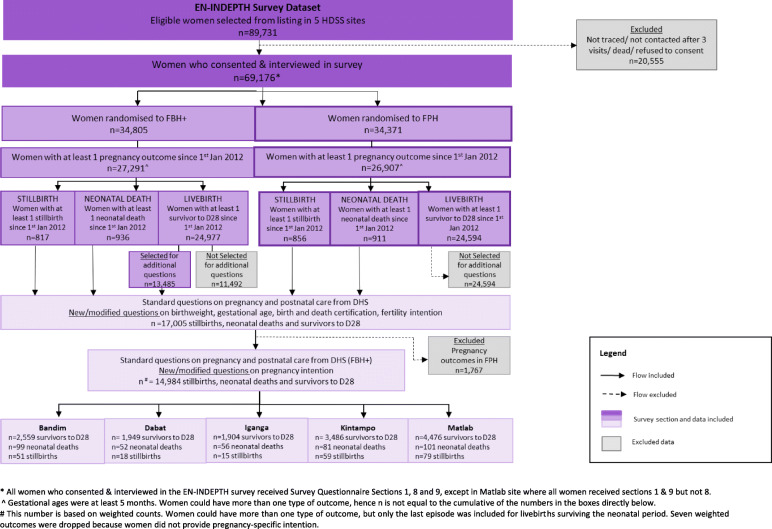
Flow diagram of EN-INDEPTH study population showing data included for pregnancy intention analyses

In this paper, we used data from the FBH+ module, which included existing questions from the DHS-7 as well as additional questions on pregnancy intention. The questions were administered to all women reporting a stillbirth (pregnancy loss after 5 months of pregnancy) or neonatal death and to a random sample of women reporting surviving live births since 1st January 2012, for their most recent pregnancy (Fig. 2, Additional file 4). Early losses at less than 5 months and miscarriages were excluded by design from these additional questions and analyses.

In this paper, we utilised two sets of questions that are widely used in surveys such as the DHS to assess pregnancy intention (Table 1A):

#### Questions assessing intention of the last pregnancy (pregnancy-specific intention)

*‘When you got pregnant with THIS BABY, did you want to get pregnant at that time?’ (Yes/No)*

*‘Did you want to have a baby later on, or did you not want any children?’ (Later/No more or None)*

A pregnancy reported as wanted now is classified as ‘wanted’, one wanted later is classified as ‘mistimed’ and one not wanted at all is classified as ‘unwanted’.

#### Questions assessing desired family size and pregnancy intention (desired-versus-actual family size)

*‘If you could go back to the time you did not have any children and could choose exactly the number of children to have in your whole life, how many would that be?’ (None/Number/Other); asked to women with at least one child at time of survey*

*‘If you could choose exactly the number of children to have in your whole life, how many would that be?’ (None/Number/Other); asked to women with no children at time of survey*

These questions aim to measure a woman’s desired family size over her reproductive lifespan. By comparing desired family size with actual number of surviving children, a woman’s intention for her last pregnancy can be ascertained; any pregnancy that occurs after the desired size has been reached is classified as ‘undesired’. For example, if a woman reported desired family size as four and had five living children at the time of survey, the last child is classified as ‘undesired’, while if she had four or fewer living children at the time of survey, the last pregnancy is regarded as ‘desired’. This question was asked in all sites, except in Matlab, Bangladesh, as a recent study investigated these topics at the site [23].

It is expected that a woman who reports her last pregnancy as unwanted in the first set of questions has reached or already exceeded her desired family size at the time of survey. So, while the questions are aimed to capture different aspects of pregnancy preferences, both methods discussed in this paper assess a woman’s attitudes towards her last pregnancy outcomes.

#### Additional questions

We asked additional questions on contraceptive use, perceived importance of delaying the last pregnancy at the time of conception, and whether or not they had considered terminating the pregnancy (Table 1B). These questions were derived from a prospective study conducted in Kenya and Bangladesh (2016–2018) aimed at understanding contraceptive non-use [23]. The study reviewed existing literature and more than 30 questionnaires on fertility preferences and reasons for non-use of family planning, and then developed a questionnaire in consultation with experts.

### Data analysis

The sample was weighted to account for the different probabilities of a pregnancy outcome being selected to ensure the sample was representative of the pregnancy outcomes that occurred to the interviewed women in the sites (Additional file 5). Weighted analyses were performed in Stata 15.1 using the svyset command.

#### Objective 1: completeness and data quality of questions in measuring pregnancy intention

To investigate completeness, the frequencies and proportions of missing data, ‘don’t know’ and ‘other’ responses were obtained for the questions. Proportions of unwanted and undesired pregnancies were obtained using two assessments (pregnancy-specific intention and desired-versus-actual family size) and then compared with the additional questions using cross-tabulations, chi-squared tests and descriptive comparisons for data quality. The level of agreement between the two sets of pregnancy intention questions was assessed. Categories from both the pregnancy-specific intention and desired-versus-actual family size sets of questions were also cross-tabulated against respondents’ reported future pregnancy intention to assess the level of agreement; it is expected that a woman who reported the last pregnancy outcome as unwanted or undesired would not want to have another child in the future. Bivariate and multivariable logistic regression were used to test the association between pregnancy intention and future pregnancy intention.

#### Objective 2: association between unintended pregnancies and maternal health care and adverse pregnancy outcomes

The associations between pregnancy intention and care-seeking and adverse outcomes were assessed for each of the two sets of pregnancy intention questions using bivariate and multiple logistic regression. For care-seeking, only live births surviving the neonatal period were included to account for reverse causality as high-risk pregnancies ending in stillbirth or neonatal death are more likely to require more complex obstetric care where this care is available [24–26]. Associations between pregnancy intention and number of ANC visits, timing of first ANC visit, place of delivery, stillbirths, neonatal deaths and low birthweight were also assessed. For low birthweight, only records with available birthweight data were included in the analysis. Biks et al. provide further information on birthweight measurement within the EN-INDEPTH study [27].

## Results

### Overall

Information on pregnancy intention was collected for the weighted sample of 14,984 most recent pregnancy outcomes since 1st January 2012. Of these, 14,373 were live births that survived the neonatal period, 390 were neonatal deaths and 221 were stillbirths (Fig. 2). In all sites, most women were aged 25–34 years and were married at the time of interview. Mean maternal age ranged from 27.8 years in Matlab to 32.2 years in Kintampo. Highest level of education and maternal health care utilisation for the last pregnancy varied by site. The overall mean reported desired family size was 4.4 children, and over 60% of respondents in IgangaMayuge and 44.3% in Kintampo reported a desired family size of five or more children. The overall median time between the women’s delivery dates and the dates they were interviewed was 26 months, with a range of 22–29 months across sites (Table 2).

#### Objective 1: completeness and data quality of questions in measuring pregnancy intention

##### Completeness of questions

Missing data were < 2% in all questions (Tables 1A and 1B). Desired family size questions, the question on preferred waiting time for the last pregnancy outcome, and two additional questions had a ‘don’t know’ option. Of these, a relatively high proportion of ‘don’t know’ responses (≥ 10%) was obtained in questions that required numerical responses: an existing question on how much longer respondents intended to wait before becoming pregnant (15.2% ‘don’t know’), and two desired family size questions posed to women with children (10.7%) and to women with no children (11.9%). Cumulatively, 12.1% of responses to the desired family size questions were ‘don’t know’ (weighted total; unweighted total was 10.7%). These were excluded in analyses on prevalence of undesired pregnancy and its associations with outcomes.

##### Prevalence estimates of unwanted and undesired pregnancies using the two sets of questions

Table 3 shows the prevalence estimates of pregnancy intention using the pregnancy-specific intention of the last pregnancy outcomes and desired family size questions. Prevalence of unwanted pregnancy was 4.1% and unintended pregnancy 19.5% (unwanted plus mistimed pregnancies) using the pregnancy-specific intention questions and 25.2% for undesired pregnancies using the desired-versus-actual family size assessment.

**Table 3:** Prevalence estimates of pregnancy intention using pregnancy-specific intention and desired-versus-actual family size assessments, EN-INDEPTH study

	Bandim *n* (%)	Dabat *n* (%)	IgangaMayuge *n* (%)	Kintampo *n* (%)	Matlab *n* (%)	Overall *n* (%)
Pregnancy-specific intendedness (*n* = 14,984)						
Unwanted	73 (2.7)	54 (2.7)	76 (3.9)	83 (2.3)	333 (7.1)	619 (4.1)
Mistimed	660 (24.4)	170 (8.4)	279 (14.1)	625 (17.2)	576 (12.4)	2310 (15.4)
Wanted	1975 (72.9)	1795 (88.9)	1620 (82.0)	2918 (80.5)	3748 (80.5)	12,055 (80.5)
Pregnancy-specific intendedness (*n* = 14,984)						
Unintended (unwanted + mistimed)	734 (27.1)	224 (11.1)	355 (18.0)	708 (19.5)	908 (19.5)	2929 (19.5)
Intended (wanted)	1975 (72.9)	1795 (88.9)	1620 (82.0)	2918 (80.5)	3748 (80.5)	12,055 (80.5)
Desired-versus-actual family size (*n* = 9033)						
Undesired	543 (25.8)	673 (37.7)	331 (17.0)	729 (22.9)	na	2276 (25.2)
Desired	1565 (74.2)	1113 (62.3)	1620 (83.0)	2459 (77.1)	na	6757 (74.8)

*na* not applicable

##### Data quality––level of agreement between current and additional questions, and future fertility desire

**Fig. 3:**
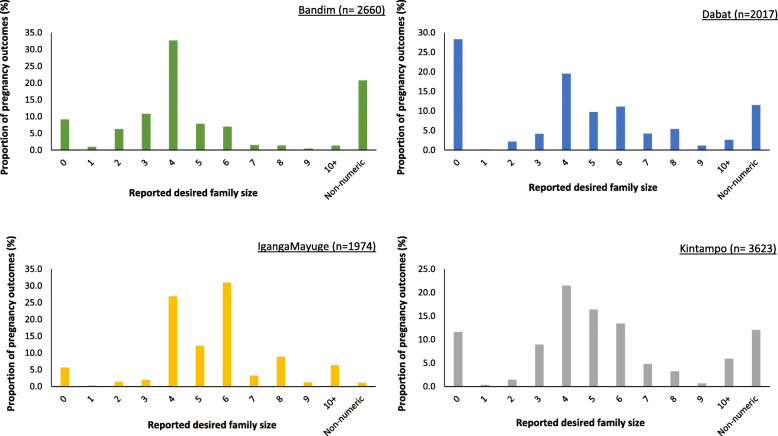
Distribution of reported desired family size by HDSS site, EN-INDEPTH study

More than a quarter (28.3%) of respondents in Dabat, and 5.6–11.6% in other sites, reported a desired family size of zero (Fig. 3). High proportions of wanted and mistimed pregnancies, using the pregnancy-specific intention questions, were also categorised as desired pregnancies, using the desired family size question: 74.9% and 82.2%, respectively (Table 4A). Similarly, 70.9% of pregnancies reported as unwanted using the pregnancy-specific intention questions were categorised as ‘undesired’ when using the desired family size question. However, the distributions of reports were significantly different, suggesting disagreement in classification of unwanted pregnancies by the two methods (*p* < 0.0001): about a quarter of (25.1%) of ‘wanted’ pregnancies, as per pregnancy-specific intention, were classified as ‘undesired’ when calculated from desired-versus-actual family size and 29.2% of ‘unwanted’ as ‘desired’.

**Table 4A:** Comparison of prevalence estimates for pregnancy-specific intention and desired-versus-actual family size for data quality (*n* = 9033)

Pregnancy-specific intention	Desired-versus-actual family size			
	Undesired (*n* (%))	Desired (*n* (%))	Overall (*n* (%))	*p* value
Unwanted *n* (%)	187 (70.9)	77 (29.2)	264 (100)	*p* < 0.0001
Mistimed *n* (%)	276 (17.8)	1270 (82.2)	1546 (100)	
Intended *n* (%)	1813 (25.1)	5410 (74.9)	7223 (100)	

Four sites. Matlab was excluded as data were not collected on desired family size

Women responding that their pregnancy was ‘unwanted’ or ‘mistimed’ for the pregnancy-specific intention were asked the additional questions (*n* = 2929). Over 90% of the respondents reported that it was ‘very important’ or ‘somewhat important’ to avoid/delay their last pregnancy (Table 4B), although more than half of these women (58.6%) reported not using any contraception at the time, with around 70% in three sites: IgangaMayuge (70.1%), Bandim (71.5%) and Kintampo (71.9%) (Table 4C). For the pregnancy-specific intention questions, a higher proportion of women with ‘unwanted’ pregnancies were using contraception at the time compared with women with ‘mistimed’ pregnancies (40.7% vs 27.7%, respectively) (*p* < 0.0001). For the desired-versus-actual family size assessment, the proportion of women who reported using contraception at the time did not differ greatly between its two categories: 33.5% for ‘undesired’ pregnancies and 29.3% for ‘desired’ pregnancies (*p* = 0.157) (Table 4B). Lastly, similar proportions of women with ‘unwanted’ and ‘mistimed’ pregnancies had considered termination (19.4% vs 19.9%, respectively). A higher proportion of women with ‘desired’ pregnancies had considered termination than women with ‘undesired’ pregnancies (21.7% vs 18.6%, respectively) using the desired-versus-actual family size assessment but there was no significant difference.

**Table 4B:** Additional questions versus the two assessments (pregnancy-specific intention and desire-versus-actual family size) among unintended pregnancies

Existing questions	Additional questions												
	Perceived importance of avoiding/delaying the last pregnancy *n* (%)					Contraception usage at the time *n* (%)				Consideration of termination *n* (%)			
	Very important	Somewhat important	Not at all important	Overall	*p* value	Yes	No	Overall	*p* value	Considered	Not Considered	Overall	*p* value
Pregnancy-specific reporting (*n* = 2021)					0.39				0.0001				0.88
Unwanted *n* (%)	243 (84.8)	27 (9.6)	16 (5.6)	286 (100)		116 (40.7)	170 (59.3)	286 (100)		56 (19.4)	231 (80.1)	286 (100)	
Mistimed *n* (%)	1435 (82.7)	212 (12.2)	88 (5.1)	1735 (100)		480 (27.7)	1254 (72.3)	1735 (100)		344 (19.9)	1390 (80.2)	1735 (100)	
Desired-versus-actual family size (*n* = 1810)					0.26				0.16				0.25
Undesired *n* (%)	374 (80.8)	59 (12.7)	30 (6.6)	463 (100)		155 (33.5)	308 (66.5)	463 (100)		86 (18.6)	376 (81.4)	463 (100)	
Desired *n* (%)	1132 (84.0)	154 (11.4)	61 (4.5)	1347 (100)		395 (29.3)	952 (70.7)	1347 (100)		293 (21.7)	1054 (78.3)	1347 (100)	

Only women with unwanted and mistimed pregnancies were asked these additional questions (importance of avoiding pregnancy, use of contraception before pregnancy and consideration of termination). Four sites. Matlab was excluded as data were not collected on desired family size

**Table 4C:** Distribution of responses to the additional pregnancy-specific intention questions by HDSS site, EN-INDEPTH study (*n* = 2929)

	Bandim *n* (%)	Dabat *n* (%)	IgangaMayuge *n* (%)	Kintampo *n* (%)	Matlab *n* (%)	Overall *n* (%)
Perceived importance of avoiding/delaying the last pregnancy						
Very important	680 (92.7)	147 (65.8)	273 (76.9)	577 (81.6)	462 (50.9)	2140 (73.1)
Somewhat important	27 (3.6)	68 (30.5)	61 (17.1)	83 (11.8)	405 (44.5)	644 (22.0)
Not at all important	27 (3.7)	8 (3.7)	21 (6)	47 (6.7)	42 (4.6)	146 (5.0)
Contraception usage at the time						
Yes	209 (28.5)	82 (36.4)	106 (29.9)	199 (28.1)	615 (67.7)	1212 (41.4)
No	524 (71.5)	142 (63.6)	249 (70.1)	293 (71.9)	509 (32.3)	1718 (58.6)
Consideration of termination						
Considered	131 (17.9)	18 (7.8)	82 (23.1)	169 (23.9)	333 (36.7)	733 (25.0)
Not considered	602 (82.1)	207 (92.2)	273 (76.9)	539 (76.1)	575 (63.3)	2196 (75.0)

Only women with unwanted and mistimed pregnancies were asked these additional questions

**Table 4D:** Future pregnancy intention of respondents by reported pregnancy-specific intention and desired-versus-actual family size categories

	Want more children *n* (%)	No more children *n* (%)	Cannot get pregnant *n* (%)	Undecided *n* (%)	Overall *n* (%)	*p* value
Pregnancy-specific intention (*n* = 10,222)						*p* < 0.0001
Unwanted	20 (7.4)	238 (86.4)	4 (1.5)	13 (4.7)	276 (100)	
Mistimed	1237 (72.3)	305 (17.8)	1 (0.07)	168 (9.8)	1710 (100)	
Intended	5706 (69.3)	1662 (20.2)	72 (0.9)	796 (9.7)	8236 (100)	
Desired-versus-actual family size (*n* = 8939)						*p* < 0.0001
Undesired	1006 (45.1)	917 (41.1)	30 (1.4)	277 (12.4)	2230 (100)	
Desired	5082 (75.7)	1078 (16.1)	42 (0.6)	507 (7.6)	6709 (100)	

Four sites. Matlab was excluded as data were not collected on desired family size

**Fig. 4A:**
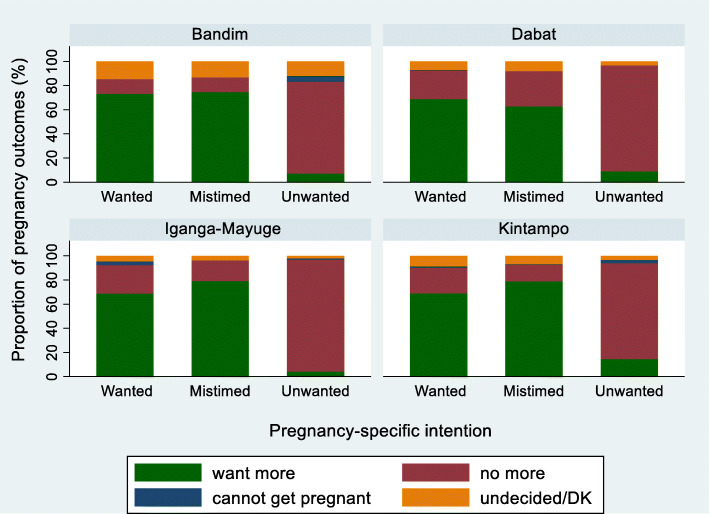

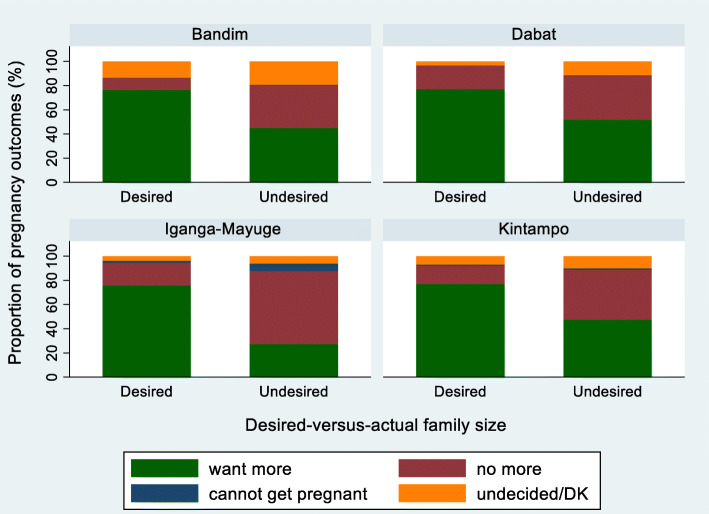
Future fertility preference by HDSS site and pregnancy-specific intention, EN-INDEPTH study

It was expected that a woman who reported the last pregnancy outcome as ‘unwanted’ or ‘undesired’ would not want to have another child in the future. Thus, the level of the agreement between the pregnancy intention of the last pregnancy outcomes and future pregnancy intention was assessed. In the pregnancy-specific intention questions, 86.4% of women with ‘unwanted’ pregnancies reported wanting no more children in the future, while 12.1% wanted more or were undecided (Table 4D); these proportions were similar across sites (Fig. 4A). In contrast, of the women with last pregnancies categorised as ‘undesired’ by desired-versus-actual family size, 45.1% reported wanting more children later (Table 4D); these proportions were also similar across sites, although slightly lower in IgangaMayuge (Fig. 4B). Despite the disagreement shown in Table 4D, overall, for the two sets of questions, women with ‘unwanted’ and ‘undesired’ pregnancies were significantly less likely to want more children in the future compared with women with ‘wanted’ or desired pregnancies (aOR 0.43 (95% CI 0.36–0.51) and aOR 0.32 (95% CI 0.27–0.37), respectively) (Additional file 6).

#### Objective 2: association between pregnancy intentions and maternal health care and adverse pregnancy outcomes

##### Associations with maternal health care utilisation

After adjusting for potential confounders, pregnancy intention using the pregnancy-specific intention questions was associated with the number and timing of ANC visits, but not place of delivery (Table 5). The odds of having 4+ ANC visits were 27% lower in ‘unintended’ compared to ‘intended’ pregnancies (aOR 0.73 (95 %CI 0.64–0.83)) and of having a first ANC visit in the first trimester 29% lower (aOR 0.71 (95% CI 0.63–0.79)) (Table 5). Similar results were also found when the pregnancy-specific intention questions were classed as ‘unwanted’ vs ‘mistimed’ plus ‘intended’ (Additional file 6). There was no association between pregnancy intention and reported maternal health care seeking using the desired-versus-actual family size assessment. Additional file 6 shows distributions of pregnancy-specific intention and desired-versus-actual family size by maternal health care utilisation.

**Table 5:** Bivariate and multivariable logistic regression for pregnancy intention and maternity care utilisation, EN-INDEPTH study

	No. of ANC visits (4+) *n*^1^ = 13,314	Timing of first ANC visits (1st trimester) *n*^1^ = 14,110	Place of delivery (health facility) *n*^1^ = 12762
	Crude OR (95% CI)	Crude OR (95% CI)	Crude OR (95% CI)
Pregnancy-specific intention			
Wanted	Ref.	Ref.	Ref.
Mistimed	1.03 (0.91–1.17)	0.88 (0.78–1.00)	1.15 (1.01–1.32)
Unwanted	0.62 (0.50–0.76)	0.60 (0.48–0.76)	0.74 (0.59–0.92)
Pregnancy-specific intention			
Intended (wanted)	Ref.	Ref.	Ref.
Unintended (unwanted + mistimed)	0.92 (0.82–1.03)	0.82 (0.73–0.91)	1.05 (0.94–1.18)
Desired-versus-actual family size			
Desired	Ref.	Ref.	Ref.
Undesired	0.63 (0.56–0.71)	0.76 (0.67–0.87)	0.57 (0.50–0.64)
	Adjusted OR (95% CI)	Adjusted OR (95% CI)	Adjusted OR (95% CI)
Pregnancy-specific intentions			
Intended (wanted)	Ref.	Ref.	Ref.
Unintended (unwanted + mistimed)	0.73 (0.64–0.83)	0.71 (0.63–0.79)	0.89 (0.78–1.02)
Desired-versus-actual family size			
Desired	Ref.	Ref.	Ref.
Undesired	0.93 (0.79–1.09)	0.92 (0.79–1.06)	1.00 (0.86–1.17)

Five sites. Includes surviving live births only. Adjusted for woman’s age, education, wealth quintile, number of living children at conception and HDSS site

^1^ For pregnancy-specific intention in the adjusted analyses, *n* = 13,312 for no. of ANC visits, 14,109 for timing of ANC and 12,761 for place of delivery. For desired-versus-actual family size in both the crude and adjusted analyses, *n* = 7816 for no. of ANC visits, 8504 for timing of first ANC and 8222 for place of delivery

##### Associations with adverse pregnancy outcomes

Associations were found between pregnancy intention and stillbirth and neonatal death but not with low birthweight, using the pregnancy-specific assessment (Table 6). Women with unintended pregnancies were 43% less likely to report a stillbirth compared with women with intended pregnancies (aOR 0.57 (95% CI 0.44–0.73)), and 21% less likely to report a neonatal death (aOR 0.79 (95% CI 0.64–0.96)). There were no associations with any of the adverse outcomes using the desired-versus-actual family size assessment. Additional file 6 shows distributions of pregnancy-specific intention and desired-versus-actual family size by pregnancy outcomes.

**Table 6:** Bivariate and multivariable logistic regression for pregnancy intention and stillbirths, neonatal deaths and birthweight

	Stillbirths *n*^1^ = 14,984	Neonatal deaths *n*^1^ = 14,763	Low birthweight *n*^1^ = 7973
	Crude OR (95% CI)	Crude OR (95% CI)	Crude OR (95% CI)
Pregnancy-specific intention			
Wanted	Ref.	Ref.	Ref.
Mistimed	0.57 (0.43–0.76)	0.74 (0.60–0.92)	0.95 (0.75–1.20)
Unwanted	0.77 (0.48–1.21)	0.99 (0.70–1.41)	1.43 (0.98–2.09)
Pregnancy-specific intention			
Intended (wanted)	Ref.	Ref.	Ref.
Unintended (unwanted + mistimed)	0.61 (0.48–0.79)	0.80 (0.66–0.96)	1.04 (0.85–1.27)
Desired-versus-actual family size			
Desired	Ref.	Ref.	Ref.
Undesired	0.81 (0.60–1.08)	0.95 (0.77–1.17)	0.96 (0.69–1.33)
	Adjusted OR (95% CI)	Adjusted OR (95% CI)	Adjusted OR (95% CI)
Pregnancy-specific intention			
Intended (wanted)	Ref.	Ref.	Ref.
Unintended (unwanted + mistimed)	0.57 (0.44–0.73)	0.79 (0.64–0.96)	1.19 (0.96–1.48)
Desired-versus-actual family size			
Desired	Ref.	Ref.	Ref.
Undesired	0.76 (0.55–1.05)	0.94 (0.74–1.19)	1.00 (0.68–1.48)

Five sites. Adjusted for woman’s age, education, wealth quintile, number of living children at conception, gender of child, single/multiple births and HDSS site (gender and single/multiple births were not included in the model for stillbirths as these data were not collected for stillbirths in the Full Birth History module).

^1^ For stillbirths, *n*=9033 for desired-versus-actual family size. For neonatal deaths, *n* = 8921 and 8916 for desired-versus-actual family size in the crude and adjusted analyses, respectively, and 14,751 for the pregnancy-specific assessment in the adjusted analyses. For birthweight, *n* = 4634 and 4616 for desired-versus-actual family size in the crude and adjusted analyses, respectively, and 7937 for the pregnancy-specific questions in the adjusted analyses

## Discussion

This paper is among the first to evaluate two widely used sets of household survey questions regarding pregnancy intention and to report on associations of these with stillbirth in any LMIC setting, and neonatal death in sub-Saharan Africa. Since survey data are crucial for monitoring maternal and newborn health for more than two-thirds of the world’s births, this paper answers an important question regarding assessment of pregnancy intention in LMICs.

### Completeness and data quality of questions in measuring pregnancy intention

In our comparison of completeness and data quality, we found challenges regarding desired-versus-actual family size questions. A total of 12.1% of the responses were excluded due to ‘don’t know’/undecided responses. Desired family size is an abstract concept and in high-fertility populations, as in our study sites, there may be an inability or unwillingness to provide a numerical answer for desired number of children if reproductive choice and agency are not yet fully accepted [28]. However, analysis of the DHS data from 32 countries showed that women’s provision of non-numeric responses to the desired family size question declined over a 19-year period (1993–2011) [28]; additionally, this study reported that as fertility rates declined, the proportion of women giving non-numeric responses also decreased. Knowledge about, and use of, contraception as well as level of education were inversely related to providing non-numeric responses where a numerical response is required [28].

Where numeric responses are provided, it is possible that women may adjust their desired number of children to match their actual number of children. Though assessment of desired-versus-actual family size is widely used by demographers to calculate aggregate wanted fertility rates [29], its utility in identifying individual undesired pregnancies was weak in our study population, and there were inconsistencies between assessment of desired-versus-actual family size and future childbearing preferences. Reported desire for another child is generally acknowledged to be the most robust assessment of fertility preferences [30]. Within our study, nearly half (45%) of women who had achieved or exceeded their desired family size at the time of conception of their most recent pregnancy nevertheless reported wanting another child (Table 4D), indicating a mismatch between the sets of questions. Similarly, we found few differences in perceived importance of avoiding or delaying the pregnancy, in contraceptive use, or in consideration of termination, between women with calculated desired and undesired pregnancies, though this lack of difference may be partly because the questions were only asked for women who stated that their most recent pregnancy outcomes were unintended (Table 4B). Lastly, the percentage of respondents who reported desired family sizes of zero was high in in Dabat (28%) (Fig. 3), which suggests that the survey question may not have been as well understood as intended. Given these points, further research should consider the challenges of asking women to provide numeric responses regarding desired family size.

The pregnancy-specific intention questions also presented some challenges. Though there was higher agreement with future childbearing desires (Table 4D), ultimately women may be reluctant to report a pregnancy as unwanted. Only 4.1% of pregnancies were classified as unwanted, which is low in view of the appreciable proportions of women who reported wanting no more children in the future and the moderate level of contraceptive use in the study sites. In societies where childbearing is highly valued, however, this reluctance may be unsurprising and the high value traditionally placed on children in many sub-Saharan African settings may act to outweigh or offset any previous intentions to avoid or delay births [8, 10]. Longitudinal studies in Senegal, Nigeria, Malawi and Kenya found that only small proportions of women who wanted no more children at baseline retrospectively classified a subsequent birth as unwanted [31].

In our study, it was more common for pregnancies to be classified as mistimed than unwanted, and 15% overall were reported as mistimed. This tendency appears strongest in Bandim where 24% of pregnancy events were reported as mistimed compared with just 2.7% as unwanted (Table 3). Though mistimed pregnancies were less likely to be associated with contraceptive use than unwanted pregnancies, this difference disappears when looking at perceived importance of delaying or avoiding a pregnancy or evoking thoughts of termination (Table 4B). The proportions of women who attached great importance to the desire to delay or avoid were almost identical for those with mistimed (82.7%) and unwanted (84.8%) pregnancies and similar for consideration of termination (19.9% and 19.4%). Our results are consistent with a large body of evidence that spacing of children is a crucial consideration intricately linked to many aspects of social life in most of sub-Saharan Africa, and short spacing between births tends to invite social criticism [32]. More recently, it has been shown that postponement of births, to delay pregnancy for an indefinite time until conditions are conducive, is an important element of reproductive culture in Africa [33]. Under this perspective, the distinction between mistimed and unwanted pregnancies become blurred.

Pregnancy intention is such a complex concept that major advances in its measurement and understanding of its implications for health and welfare are unlikely to be achieved without the application of prospective survey designs and multi-item scales, such as the London Measure of Unplanned Pregnancy (LMUP), a six-item scale which measures the degree of pregnancy intention and which has been validated in a number of low-income settings [34–37]. Two severe barriers stand in the way of progress. First, evidence on pregnancy intention will continue to come primarily from cross-sectional surveys because large, representative prospective studies are so expensive and time-consuming. Second, these surveys are likely to be multi-purpose, such as the DHS, with many competing interests and questions, making it improbable that space will be found for more ambitious pregnancy intention measures such as LMUP.

The three additional questions added in this study had some value. Perceived importance of delay/avoidance may be the most promising question in terms of level of agreement (only less than 6% of women with ‘unwanted’ and mistimed pregnancies did not perceive delay or avoidance as important). Contraceptive use may likely be influenced by accessibility issues and consideration of termination by socio-cultural factors, personal values and reporting bias [38]. Given this, these additional questions are not the only possible succinct additions to the standard DHS approach. Our results from the additional questions demonstrate that lack of control of pregnancy timing and spacing is not a trivial matter for women and that pregnancy timing is an equal concern to limitation. The results from the question on contraception, which is a component of LMUP, revealed the substantial gap between desires and behaviour, and has obvious programme implications.

### Association between unintended/unwanted/undesired pregnancies and maternal health care and adverse pregnancy outcomes

We showed an association between pregnancy intention and timing and frequency of ANC visits. Women who reported their pregnancy as intended had more frequent and earlier ANC visits. This is consistent with previous studies [39–42] and likely indicates positive health-seeking behaviour amongst mothers who had intended to become pregnant. Using the pregnancy-specific intention questions, we found some evidence of an association between pregnancy intention and stillbirths and neonatal deaths, with women with unintended births less likely to report these outcomes. No such association was found when using the desired-versus-actual family size assessment. Previous evidence on this topic is mixed. In line with our findings, Smith-Greenaway et al. found that pregnancies resulting in neonatal death were less likely to be reported as unintended, in their study of DHS data from 31 sub-Saharan African countries [43]. Longitudinal studies in Ghana and Malawi found no association between pregnancy wantedness and child survival [8, 10], while Hall et al. found some evidence of reduced risk of stillbirth for intended pregnancies, but no association with neonatal mortality, miscarriage and low birthweight nor with a composite measure comprising of all four outcomes [17]. However, Singh and Chalansani identified increased risk of neonatal mortality amongst unwanted pregnancies in Bangladesh and India [13, 44], while in their study of pregnancy intendedness in Ethiopia, Assefa et al. found increased odds of pregnancy loss, defined as miscarriage, induced abortion and stillbirth, amongst unintended pregnancies [16]. Other studies have also reported increased likelihood of other outcomes including premature rupture of membranes, preterm delivery and poor child outcomes, for unwanted pregnancies [45, 46].

Underreporting of neonatal deaths and stillbirths may have influenced our results to some extent. As such, generalisability of findings to other sites may be diminished [3]. As discussed, women may be reluctant to report a child, particularly one who has died, as unwanted or undesired, and may revise their intention or desired family size following conception or birth. Additionally, persistent stigma associated with neonatal deaths and, in particular, stillbirths across many LMIC and HIC settings can undermine collection and analysis of survey data [47]. Detailed qualitative analysis of barriers to reporting stillbirths and neonatal deaths within household surveys are reported elsewhere in this series [48], and we highlight this here again as an important gap in data collection for maternal and newborn health.

### Strengths and limitations

This paper reports on pregnancy intention and related outcomes from five HDSS sites in sub-Saharan Africa and Asia, utilising data on almost 15,000 stillbirths, neonatal deaths and live births. This research provides a valuable contribution to assessing pregnancy intention in LMICs, including an in-depth analysis of utility for two commonly used methods of assessing pregnancy intention in surveys plus additional questions. We have also reported on associations between pregnancy intention, health care utilisation and adverse pregnancy outcomes, with important programmatic consequences. Through our analysis of pregnancy intention and stillbirths, which has received less attention in previous studies, this paper provides a valuable contribution to stillbirth research.

Limitations include a cross-sectional study design, retrospective data collection, challenges with representing a nuanced understanding of pregnancy intention, restrictions on some analysis of stillbirths and neonatal deaths, and a high proportion of missing birthweight data. Firstly, and importantly, our cross-sectional survey design, as in the DHS, makes it difficult to establish pregnancy intentions prior to birth and limits assessing of causal association between pre-birth intention and stillbirth and neonatal outcomes. It is widely reported that retrospectively assessing pregnancy intention may underestimate unintended pregnancy due to reluctance to report a child as unwanted or mistimed following pregnancy and birth [15, 43, 49–52]. In addition, some women in this study were reporting on intention for pregnancies conceived up to 7 years prior to the survey, and previous evidence has shown that the longer the time since conception, the less likely a pregnancy is to be reported as unintended or unwanted [43, 53]. However, median length of recall of intention in the study appeared to be quite short; 26 months overall, with a range of just a few months (22–29 months) across sites. Secondly, the use of binary and categorical variables in this study may place limits on our understanding of pregnancy intention, as the concept is socially contextualised and may be better captured on a range or continuum of feelings or intentions. Pregnancy intention is so closely linked to sociodemographic conditions that separating and categorising intentions in meaningful ways becomes difficult [9], and broadening the definition to include multiple social and cultural understandings of pregnancy intention is important. Thirdly, in examining the association between pregnancy intention and maternal health care utilisation, we restricted analyses to live births only, as most high-risk pregnancies ending in stillbirth or neonatal death are more likely to elicit greater obstetric care and thus increase maternal health care utilisation [24–26], which may have influenced our results. Lastly, 46.8% of birthweight data were missing, and it is unclear how this may have influenced the association between pregnancy intention and low birthweight.

## Conclusion

The application of the desired-versus-actual family size approach had limited utility in identifying specific undesired pregnancies. However, responses to the pregnancy-specific questions were useful, despite a reluctance to report pregnancies as unwanted rather than mistimed, and we found these questions to be the most reliable. The additional questions to the pregnancy-specific questions showed that the subjective importance to women of mistimed and unwanted pregnancies was similar. These questions may complement current questions used in the DHS, although these are not the only possible additions. Women with unintended pregnancies may already be vulnerable, and we found that they are more likely to miss timely and frequent antenatal care, so implementation research is required as to how to improve coverage and quality of care for those women.

## Supplementary information

**Additional file 1:** Background overview of the five HDSS sites.

**Additional file 2:** Qualitative methods for Focus Group Discussions in the EN-INDEPTH study.

**Additional file 3:** STROBE guidelines checklist.

**Additional file 4:** Selection of women with a livebirth surviving the neonatal period, EN-INDEPTH survey.

**Additional file 5:** Calculation of survey weights.

**Additional file 6:** Additional results.

**Additional file 7:** Ethical approval of local Institutional Review Boards.

## Data Availability

Data sharing and transfer agreements were jointly developed and signed by all collaborating partners. The datasets generated during the current study are deposited online at https://doi.org/10.17037/DATA.00001556 with data access subject to approval by collaborating parties.

## Abbreviations

ANC: Antenatal care
DHS: Demographic and Health Surveys
ENAP: Every Newborn Action Plan
EN-INDEPTH: Every Newborn–International Network for the Demographic Evaluation of Populations and their Health
FBH+: Full birth history (+ denotes additional questions on pregnancy losses)
FGD: Focus group discussion
FPH: Full pregnancy history
HDSS: Health and Demographic Surveillance System
LMICs: Low- and middle-income countries
LMUP: London Measure of Unplanned Pregnancy
MICS: Multiple Indicator Cluster Survey
PNC: Postnatal care
SDG: Sustainable development goals
UN: United Nations
WHO: World Health Organization
