# Supplementary material for: Pregnancy intention data completeness, quality and utility in population-based surveys: EN-INDEPTH study
Source: Popul Health Metr. 2021 Feb 8;19(Suppl 1):6. doi: 10.1186/s12963-020-00227-y (PMC7869206; doi:10.1186/s12963-020-00227-y)
Supplement: Supplementary file 5 — Additional file 5: Calculation of survey weights. [file 12963_2020_227_MOESM5_ESM.docx]

## Additional file 5: Calculation of survey weights

The analysis was restricted to women interviewed in the FBH+ arm.

***Step 1:*** Calculate the probability of a woman with any pregnancy outcome (livebirth, neonatal death, stillbirth) since 1^st^ January 2012 receiving the pregnancy and birth module. For a woman with a stillbirth or neonatal death since 1^st^ January 2012 the probability of being included is 1, as all women with a neonatal death received these additional questions.

For a woman with a livebirth surviving the neonatal period, the probability of receiving the additional questions varied by HDSS site (see Annex 1). The weight was calculated as:

$$\frac{100}{\% of women in FBH+ arm completing pregnancy and birth module}$$

For example, in Bandim, the weight was calculated as 100/28.3.

***Step 2:*** The weight for each individual pregnancy outcome was calculated as the inverse of the probability of the pregnancy outcome being selected for each record. For example, if a woman had 2 neonatal deaths after 2012, only the last neonatal could be included so the individual sampling weight would be 2/1.

***Step 3:*** The weights calculated under steps 1 and 2 were normalized. First the mean weight of all the selected records was calculated. Then the weights calculated in step 2 were divided by the mean weight to estimate new weights. The mean of new weights is 1.
