# Supplementary material for: Pregnancy intention data completeness, quality and utility in population-based surveys: EN-INDEPTH study
Source: Popul Health Metr. 2021 Feb 8;19(Suppl 1):6. doi: 10.1186/s12963-020-00227-y (PMC7869206; doi:10.1186/s12963-020-00227-y)
Supplement: Supplementary file 6 — Additional file 6: Additional results. [file 12963_2020_227_MOESM6_ESM.docx]

## Additional file 6: Additional results

### **Additional file 6.1**^1^: Bivariate and multivariable logistic regression for the two assessments and future pregnancy intention, EN-INDEPTH study

|  | **Future pregnancy intention (‘want more children’)** |
| --- | --- |
|  | **Crude OR (95% CI)** |
| **Pregnancy-specific intention (n=9168)** |  |
| Wanted | Ref. |
| Mistimed | 1.18 (0.99- 1.41) |
| Unwanted | 0.025 (0.016- 0.038) |
| **Pregnancy-specific intention (n=9168)** |  |
| Intended (wanted) | Ref. |
| Unintended (unwanted + mistimed) | 0.67 (0.59- 0.78) |
| **Desired-versus-actual family size (n=8083)** |  |
| Desired | Ref. |
| Undesired | 0.23 (0.20- 0.27) |
|  | **Adjusted OR (95% CI)** |
| **Pregnancy-specific intention (n=9037)** |  |
| Intended | Ref. |
| Unintended (unwanted + mistimed) | 0.43 (0.36- 0.51) |
| **Desired-versus-actual family size (n=7976)** |  |
| Desired | Ref. |
| Undesired | 0.32 (0.27- 0.37) |

^1^ Five sites. “Cannot get pregnant” and “undecided” categories were excluded from the analyses. Adjusted for woman’s age, education, wealth quintile, gender of child, single/multiple births and HDSS site.

### **Additional file 6.2^1^**: Additional results from multivariable analyses for pregnancy-specific intention^2^ and future pregnancy intention, maternity care utilisation and adverse pregnancy outcomes

|  | **Future pregnancy intention** | **Maternity care utilisation** | | | **Adverse pregnancy outcomes** | | | |
| --- | --- | --- | --- | --- | --- | --- | --- | --- |
|  |  | **No. of ANC visits**  **n=13314** | **Timing of ANC visits n=14110** | **Place of delivery n=12762** | **Stillbirths**  **n=14984** | **Neonatal deaths**  **n=14763** | **Low birthweight**  **n=7973** |  |
|  | **Crude OR (95% CI)** | **Crude OR (95% CI)** | **Crude OR (95% CI)** | **Crude OR (95% CI)** | **Crude OR (95% CI)** | **Crude OR (95% CI)** | **Crude OR (95% CI)** |  |
| **Pregnancy-specific intention (n=9168)** |  |  |  |  |  |  |  |  |
| Wanted + mistimed | Ref. | Ref. | Ref. | Ref. | Ref. | Ref. | Ref. |  |
| Unwanted | 0.024 (0.016- 0.037) | 0.61 (0.50- 0.76) | 0.62 (0.49- 0.77) | 0.72 (0.58- 0.90) | 0.82 (0.52- 1.30) | 1.04 (0.73- 1.47) | 1.44 (0.99- 2.11) |  |
|  | **Adjusted OR (95% CI)** | **Adjusted OR (95% CI)** | **Adjusted OR (95% CI)** | **Adjusted OR (95% CI)** | **Adjusted OR (95% CI)** | **Adjusted OR (95% CI)** | **Adjusted OR (95% CI)** |  |
| **Pregnancy-specific intention (n=9168)** |  |  |  |  |  |  |  |  |
| Wanted + mistimed | Ref. | Ref. | Ref. | Ref. | Ref. | Ref. | Ref. |  |
| Unwanted | 0.034 (0.021- 0.057) | 0.72 (0.58- 0.91) | 0.71 (0.56- 0.90) | 0.91 (0.70- 1.19) | 0.61 (0.38- 0.98) | 1.08 (0.74- 1.56) | 1.41 (0.93- 2.14) |  |

^1^ Four sites

^2^ ‘Wanted’ and ‘mistimed’ categories were combined for pregnancy-specific intention

### **Additional file 6.3^1^:** Pregnancy-specific intention and desired-versus-actual family size by maternity care utilisation for surviving livebirths, EN-INDEPTH study

|  | **No. of ANC visits n (%)**  **n^1^= 13313** | | | | **Timing of first ANC n (%)**  **n^1^=14110** | | | **Place of delivery n (%)**  **n^1^=12762** | | |
| --- | --- | --- | --- | --- | --- | --- | --- | --- | --- | --- |
|  | **0**  **n (%)** | **1-3**  **n (%)** | **4+**  **n (%)** | **P value** | **1st trimester**  **n (%)** | **later or none**  **n (%)** | **P value** | **Home**  **n (%)** | **Health facilities**  **n (%)** | **P value** |
| **Pregnancy-specific intention** |  |  |  | <0.0001 |  |  | p<0.0001 |  |  | 0.0018 |
| Unwanted | 58 (10.2) | 211 (36.9) | 303 (53.0) |  | 168 (28.4) | 424 (71.6) |  | 212 (43.0) | 281 (57.0) |  |
| Mistimed | 115 (5.6) | 598 (29.1) | 1345 (65.3) |  | 803 (36.6) | 1388 (63.4) |  | 657 (32.6) | 1360 (67.4) |  |
| Wanted | 839 (7.8) | 2935 (27.5) | 6911 (64.7) |  | 4487 (39.6) | 6841 (60.4) |  | 3670 (35.8) | 6582 (64.2) |  |
| **Pregnancy-specific intention** |  |  |  | 0.02 |  |  | 0.0003 |  |  | 0.39 |
| Unintended (Unwanted + mistimed) | 173 (6.6) | 807 (30.8) | 1647 (62.7) |  | 971 (34.9) | 1812 (65.1) |  | 869 (34.6) | 1641 (65.4) |  |
| Intended(Wanted) | 839 (7.8) | 2935 (27.5) | 6911 (64.7) |  | 4487 (39.6) | 6841 (60.4) |  | 3670 (35.8) | 6582 (64.2) |  |
| **Desired-versus-actual family size** |  |  |  | p<0.0001 |  |  | p<0.0001 |  |  | p<0.0001 |
| Undesired | 303 (15.4) | 492 (25.1) | 1169 (59.5) |  | 1400 (65.6) | 733 (34.4) |  | 897 (42.6) | 1209 (57.4) |  |
| Desired | 356 (6.1) | 1398 (23.9) | 4098 (70.0) |  | 3782 (59.3) | 2591 (40.7) |  | 1808 (29.6) | 4308 (70.4) |  |

^1^ Five sites. For desired-versus-actual family size assessment, n=7816 for No. of ANC visits, 8504 for timing of first ANC and 8222 for place of delivery.

###

**Additional file 6.4^1^**: Pregnancy-specific intention and desired-versus-actual family size by pregnancy outcome, EN-INDEPTH study

|  | **Livebirths vs stillbirths**  **n^1^= 14984** | | | **Survived vs neonatal deaths**  **n^1^=14762** | | | **Survived vs neonatal deaths vs stillbirths**  **n^1^=14984** | | | | **Birthweight**  **n^1^=7973** | | |
| --- | --- | --- | --- | --- | --- | --- | --- | --- | --- | --- | --- | --- | --- |
|  | **Livebirths**  **n (%)** | **Stillbirths**  **n (%)** | **P value** | **Survived**  **n (%)** | **Neonatal death n (%)** | **P value** | **Survived**  **n (%)** | **Neonatal deaths n (%)** | **Stillbirths**  **n (%)** | **P value** | **Less than 2.5 kg n (%)** | **2.5kg+**  **n (%)** | **P value** |
| **Pregnancy-specific intention** |  |  | 0.0003 |  |  | 0.024 |  |  |  | 0.0002 |  |  | 0.15 |
| Unwanted | 611 (98.8) | 8 (1.2) |  | 595 (97.3) | 17 (2.7) |  | 595 (96.1) | 17 (2.7) | 8 (1.2) |  | 60 (18.7) | 259 (81.3) |  |
| Mistimed | 2289 (99.1) | 21 (0.9) |  | 2242 (97.9) | 47 (2.1) |  | 2242 (97.0) | 47 (2.0) | 21 (1.0) |  | 174 (13.2) | 1141 (86.8) |  |
| Wanted | 11862 (98.4) | 193 (1.6) |  | 11537 (97.3) | 326 (2.8) |  | 11537 (95.7) | 326 (2.7) | 193 (1.6) |  | 878 (13.9) | 5461 (86.2) |  |
| **Pregnancy-specific intention** |  |  | 0.0001 |  |  | 0.017 |  |  |  | 0.0000 |  |  | 0.72 |
| Unintended (unwanted + mistimed) | 2900 (99.0) | 29 (1.0) |  | 2837 (97.8) | 64 (2.2) |  | 2837 (96.8) | 64 (2.2) | 29 (1.0) |  | 234 (14.3) | 1400 (85.7) |  |
| Intended (wanted) | 11862 (98.4) | 193 (1.6) |  | 11537 (97.3) | 326 (2.8) |  | 11537 (95.7) | 326 (2.7) | 193 (1.6) |  | 878 (13.9) | 5461 (86.2) |  |
| **Desired-versus-actual family size** |  |  | 0.15 |  |  | 0.63 |  |  |  | 0.35 |  |  | 0.80 |
| Undesired | 2252 (99.0) | 24 (1.0) |  | 2191 (97.3) | 61 (2.7) |  | 2191 (96.3) | 61 (2.7) | 17 (1.0) |  | 85 (9.3) | 827 (90.7) |  |
| Desired | 6669 (98.7) | 88 (1.3) |  | 6479 (97.2) | 190 (2.9) |  | 6479 (95.9)) | 190 (2.8) | 94 (1.3) |  | 359 (9.6) | 3364 (90.4) |  |

^1^ Five sites. For desired-versus-actual family size, n=9033 for livebirths (survived and neonatal deaths) vs stillbirths, 8921 for survived vs neonatal deaths, 9033 for survived vs neonatal deaths vs stillbirths and 4634 for birthweight
