# Supplementary material for: Pregnancy intention data completeness, quality and utility in population-based surveys: EN-INDEPTH study
Source: Popul Health Metr. 2021 Feb 8;19(Suppl 1):6. doi: 10.1186/s12963-020-00227-y (PMC7869206; doi:10.1186/s12963-020-00227-y)
Supplement: Supplementary file 7 — Additional file 7: Ethical approval of local Institutional Review Boards. [file 12963_2020_227_MOESM7_ESM.docx]

## Additional file 7: Ethical approval of local Institutional Review Boards

| **HDSS site** | **Institutional Review Boards** | **Date** | **Number/Ref** |
| --- | --- | --- | --- |
| Bandim | Comité Nacional de Ética na Saúde | 12 June 2017 | 072/CNES/INASA/2017 |
| Dabat | Institutional Review Board, University of Gondar | 19 April 2017 | VP/RCS/05/1074/2016 |
| IgangaMayuge | Mildmay Uganda Research Ethics Committee  Uganda National Council of Science and Technology | 26 June 2017  11 October 2017 | REC REF 0305-2017  SS 4244 |
| Kintampo | Kintampo Health Research Centre, Ghana Health service  Ghana Health Services Ethics Review Committee  Kintampo Health Research Centre Institutional Ethics Committee | 14 June 2017  26 July 2017  9 August 2017 | SRC/130617  GHS-ERC:19/06/14  KHRCIEC/2017-14 |
| Matlab | Icddr,b Ethical Review Committee | 19 July 2017 | PR-17049 |
| London School of Hygiene & Tropical Medicine | London School of Hygiene & Tropical Medicine | 24 May 2017 | 12218 |
